# What is needed to improve sexual health and well-being

**DOI:** 10.2471/BLT.24.292864

**Published:** 2024-12-01

**Authors:** Manjulaa Narasimhan, Pascale Allotey

**Affiliations:** a Department of Sexual and Reproductive Health, World Health Organization, Avenue Appia 20, 1211 Geneva, Switzerland.

Transformative approaches to sexual health and well-being across the life course are essential to advance comprehensive sexual and reproductive health and rights, protection of bodily autonomy and gender justice. Much of the existing evidence views this health topic reductively – focusing on maternal and reproductive health – and views sexual health as primarily about sexually transmitted infections. Sexual health is historically overlooked, and underfunded, and sexual well-being ignored. Additional efforts are therefore needed to prioritize sexual health and well-being within the broader framework of sexual and reproductive health and rights to foster more inclusive and equitable health systems for all.^1^ This theme issue aims to spark dialogue and highlight current evidence from both health system and people-centred perspectives on sexual health and well-being.^2^

The papers in this theme issue reiterate the message that sexual health and well-being are essential to overall health^3^ over the entire life course, including for adolescents,^4^ people of reproductive age and older people.^5^ This issue also emphasizes the importance of action from policy to practice – from the growing threats of sexually transmitted infections^6^ and the dangers of sexual exploitation during conflicts,^7^ to sexual health and well-being beyond linkages to reproduction, including understanding menstrual health as an issue of sexual justice,^8^ and preparing women for perimenopause.^9^ As global understanding of sexual health and well-being still appears to be largely disease focused,^10^ a broader, people-centred focus on sexual empowerment^11^ and social and commercial determinants of sexual health is needed. Such an evolution must also support the integration of high-quality, accessible, affordable, available and acceptable self-care options for sexual health. Self-care options can be provided through telehealth, including for underserved communities, as in a mobile health intervention for HIV prevention among female sex workers.^12^

Further work in this area is needed to expand access to sexual health services and increase research on the range of sexual health experiences and needs in a broader, more diverse range of populations.^3^^,^^13^ The UNDP/UNFPA/UNICEF/WHO/World Bank Special Programme of Research, Development and Research Training in Human Reproduction, contained in the World Health Organization (WHO) Department of sexual and reproductive health and research, is the main instrument within the United Nations for promoting, conducting, evaluating and coordinating interdisciplinary research in sexual and reproductive health and rights. The programme has driven meaningful change over the past 50 years, and is further strengthening attention on sexual health and well-being across the life course, as evidenced by the multicountry findings on sexual health practices and experiences and addressing harmful norms in sexual health services.^14^

Building a strong evidence base is an important tool to fight misinformation and political opposition that have long hindered progress in sexual and reproductive health and rights more broadly, and sexual health and well-being in particular, with damaging effects on societies, individuals and marginalized communities. WHO could play a vital role in establishing a global sexual and reproductive health and rights information hub, offering fact-based resources. Initiatives could include myth-busting campaigns on social media, and partnerships with schools to integrate comprehensive sexuality education. Competency-based training for health and care workers on inclusive communication methods can further support efforts to reach diverse populations. By fostering informed, open conversations around sexual health and well-being, such initiatives can build trust and dispel harmful misconceptions.

Securing the broader realization of sexual health and well-being within the sexual and reproductive health and rights framework requires an ambitious, forward-looking strategy, grounded in robust research, that prioritizes issues such as inclusivity, combats misinformation, and uses innovations such as self-care and digital health interventions. These approaches are needed to strengthen resilience in crisis settings, where health systems are often disrupted. By strengthening the sexual health and well-being aspects of sexual and reproductive health and rights, these rights can remain an accessible, integral aspect of global health, supporting the health, well-being and autonomy of all individuals. Doing so will require broad stakeholder collaboration to tackle issues such as restrictive policies and social stigma. Funding will also be required for research on diverse sexual health priorities, including for marginalized, underserved populations. With commitment and coordination, the global health community can make strides towards a future where sexual and reproductive health and rights are universally recognized, protected and celebrated.

## References

[1] Ghebreyesus TA, Allotey P, Narasimhan M. Advancing the “sexual” in sexual and reproductive health and rights: a global health, gender equality and human rights imperative. Bull World Health Organ. 2024 Jan 1;102(1):77–8. 10.2471/BLT.23.291227 38164333 PMC10753275

[2] Narasimhan M, Gilmore K, Murillo R, Allotey P. Sexual health and well-being across the life course: call for papers. Bull World Health Organ. 2023 Dec 1;101(12):750–750A. 10.2471/BLT.23.291043

[3] Vasconcelos P, Carrito ML, Quinta-Gomes AL, Patrão AL, Nóbrega CAP, Costa PA, et al. Associations between sexual health and well-being: a systematic review. Bull World Health Organ. 2024 Dec 1;102(12):873–87D. 10.2471/BLT/2024/291565 PMC11601183 39611198

[4] Mahomed S, Bukusi E, Sikazwe I, Musoke P, Karim QA. A risk- and needs-based strategy for HIV prevention for adolescent girls and young women, WHO African Region. Bull World Health Organ. 2024 Dec 1;102(12):913–15. 10.2471/BLT/2023/291160 PMC11601182 39611197

[5] McAuliffe L, Fetherstonhaugh D. Sexual health and well-being in later life. Bull World Health Organ. 2024 Dec 1;102(12):916–18. 10.2471/BLT/2024/291576 PMC11601190 39611193

[6] Ndembi N, Folayan MO, Abdool Karim S. Mpox: an old disease poses a new threat. Bull World Health Organ. 2024 Dec 1;102(12):845–845A. 10.2471/BLT/2024/292707 PMC11601181 39619833

[7] Westendorf JK, Bian J, Daigle M, Potts A, Jennings K, Massonneau CC, et al. Sexual exploitation, abuse and harassment in humanitarian contexts. Bull World Health Organ. 2024 Dec 1;102(12):888–94. 10.2471/BLT/2024/291655 PMC11601185 39611187

[8] Logie C, Winkler IT, Narasimhan M, Bhakta A, Hutchingame T, Reynolds T, et al. Menstruation and sexual health, well-being and justice. Bull World Health Organ. 2024 Dec 1;102(12):904–06. 10.2471/BLT/2024/291159 PMC11601178 39611195

[9] Pyne Y, Burgin J, Hickey M. Towards a more accurate global picture of perimenopause. Bull World Health Organ. 2024 Dec 1;102(12):922–24. 10.2471/BLT/2024/292659 PMC11601186 39611191

[10] Owolabi O, Hopkins J, Bankrole A, Bearak JM. Progress towards sustainable development goals related to sexual health. Bull World Health Organ. 2024 Dec 1;102(12):895–903. 10.2471/BLT/2024/291163 PMC11601179 39611188

[11] Kågesten A, van Reeuwijk M, Moreau C. Improving measures of women’s and girls’ sexual empowerment. Bull World Health Organ. 2024 Dec 1;102(12):907–09. 10.2471/BLT/2024/291975 PMC11601187 39611196

[12] Mbotwa CH, Kazaura MR, Moen K, Sudfeld CR, Metta E, Leshabari MT, et al. Trial of an mHealth intervention to improve HIV prophylaxis for female sex workers, United Republic of Tanzania. Bull World Health Organ. 2024 Dec 1;102(12):852–60. 10.2471/BLT/2024/291516 PMC11601177 39611190

[13] Rahmioglu N, Zondervan K. Endometriosis: disease mechanisms and health disparities. Bull World Health Organ. 2024 Dec 1;102(12):919–21. 10.2471/BLT/2024/292660 PMC11601192 39611194

[14] Hunter E, Fine E, Black K, Henriks J, Tofail F, Morroni C, et al. Cognitive testing in 19 countries to refine WHO’s Sexual Health Assessment of Practices and Experiences. Bull World Health Organ. 2024 Dec 1;102(12):861–72. 10.2471/BLT/2023/291162 PMC11601188 39611192

